# Comprehensive transcriptomic and metabolomic profiling reveals the differences between alfalfa sprouts germinated with or without light exposure

**DOI:** 10.3389/fpls.2022.943740

**Published:** 2022-08-05

**Authors:** Kangning Zhang, He Li, Tian Zhang, Shixing Wang, Liang Liu, Xuyan Dong, Lili Cong, Hui Song, Aihua Wang, Guofeng Yang, Hongli Xie, Zeng-Yu Wang, Maofeng Chai

**Affiliations:** ^1^Key Laboratory of National Forestry and Grassland Administration on Grassland Resources and Ecology in the Yellow River Delta, College of Grassland Science, Qingdao Agricultural University, Qingdao, China; ^2^College of Food Science and Engineering, Qingdao Agricultural University, Qingdao, China

**Keywords:** alfalfa sprouts, transcriptomic analysis, metabolomic analysis, isoflavonoid biosynthesis, light exposure

## Abstract

Alfalfa sprouts are among the most nutritionally rich foods, and light exposure is a critical factor in determining their biomass and quality. However, detailed metabolic and molecular differences between yellow and green alfalfa sprouts remain unclear. In this study, comprehensive metabolomic and transcriptomic analyses were integrated to evaluate the nutrient composition of alfalfa sprouts during germination with or without light exposure. Differentially expressed genes and differentially accumulated metabolites in green and yellow alfalfa sprouts were significantly enriched in secondary metabolic pathways, such as the isoflavonoid biosynthesis pathway. Green alfalfa sprouts contained a wide variety of lipids, flavonoids, phenolic acids, and terpenoids, among which the top three upregulated were calycosin, methyl gallate, and epicatechin 3-gallate, whereas yellow alfalfa sprouts contained relatively more isoquercitrin. These results provide new insights into the nutritional value and composition of alfalfa sprouts under different germination regimes.

## Introduction

An increasing number of consumers are pursuing healthier lifestyles and are more likely to prefer green foods that are nutrient-dense or rich in particular elements. Sprouts are a good option for consumers because they are rich in biologically active substances such as antioxidants, polyphenols, vitamins, and minerals, which benefit human health and prevent various diseases (Mir et al., 2021). Sprouts are obtained from seeds germinated and grown in water or other media, and harvested before the true leaves develop (Benincasa et al., 2019). Distinct types of sprouts contain different kinds and amounts of nutrients. Chinese kale sprouts are rich in glucosinolates and their derivatives (Zeng et al., 2021). Broccoli sprouts contain isothiocyanates, a metabolite of glucosinolates, which can prevent cancer, inflammatory, and cardiovascular diseases (Yin et al., 2021). Tartary buckwheat sprouts contain high levels of rutin, a substance with antioxidant effects (Ma et al., 2021). During the germination of lentils, the melatonin content increases, and lentil sprout extracts can improve plasma melatonin concentration and attenuate plasmatic oxidative stress in rats (Rebollo-Hernanz et al., 2020). The extracts of 6-day-old quinoa sprouts have excellent antioxidant activity, which can significantly reduce both low-density and very-low-density lipoprotein levels in rats under oxidative stress, increase high-density lipoprotein concentration to ameliorate the prognosis of blood disorders, significantly reducing malondialdehyde content (Al-Qabba et al., 2020). Saponins in barley sprout hot water extract inhibit 3T3-L1 preadipocyte differentiation into adipocytes, contributing to weight loss (Kim et al., 2021).

Alfalfa has been named “the father of all food” and “the queen of forages” since ancient times for its nutrient-dense contents and beneficial health effects (Ibrahim et al., 2020). For instance, apigenin, a substance extracted from alfalfa sprouts, can revert thyroid function in hyperthyroid rats back to normal (Ibrahim et al., 2020). Adding alfalfa sprouts to diets improved the fat and fatty acid contents, and phytochemical characteristics in rabbit meat (Dal Bosco et al., 2015), reducing the plasma cholesterol content in hen eggs (Mattioli et al., 2016). However, the nutritional value of alfalfa sprouts has not yet been comprehensively explored. In recent years, research on alfalfa sprouts has mainly focused on microbial contamination (Fedio et al., 2012; Brankatschk et al., 2014; Keshri et al., 2019; Jang et al., 2021; Zheng et al., 2021) and the related evaluation of physical (Michalczyk et al., 2019; Mohammad et al., 2019), chemical (Machado-Moreira et al., 2021; Zhang et al., 2021), and biological (Fong et al., 2017; Kim et al., 2020) controls. Studies on the nutritional quality of alfalfa sprouts have mainly investigated the dynamic changes in their nutritional content, composition, and antioxidant properties, during germination under different conditions. Studies have shown that antioxidant activity and levels of Ca, Se, Fe, K, Ni, total polyamines, and total polyphenols increase during alfalfa seed germination (Taraseviciene et al., 2019; Chiriac et al., 2020a,b; Cigic et al., 2020). Alfalfa sprouts treated with a range of acoustic frequencies showed higher levels of L-ascorbic acid and flavonoids, and higher activity of superoxide dismutase (Kim et al., 2017, 2020). High concentrations of CO_2_ promoted the accumulation of carbohydrates, proteins, fats, and fiber, and increased the contents of vitamins, phenols, flavonoids, and minerals (Almuhayawi et al., 2021). Different light intensities and qualities also had different effects on the growth and total polyphenol, ascorbic acid, chlorophyll, and β-carotene contents of alfalfa sprouts (Kwack et al., 2015; Fiutak et al., 2019). Other studies have focused on adding specific elements to culture media to obtain alfalfa sprouts enriched in specific elements such as iron, selenium, and magnesium (Funes-Collado et al., 2013; Park et al., 2014; Przybysz et al., 2016a,b).

Light, one of the most important environmental factors for plants, affects the nutritional contents and compositions of sprouts (Zhang et al., 2020). Different types of light sources and intensities caused different enrichment patterns of light-induced stilbenes and total phenol contents in peanut sprouts (Zhu et al., 2021). Light treatment increased the levels of vitamin C and those of various pigment compounds in radish, soybean, mung bean, and pumpkin sprouts, as well as the soluble sugar content, including D-glucose, D-fructose, and sucrose, in pumpkin sprouts (Mastropasqua et al., 2020).

Alfalfa sprouts can be grown as either yellow or green sprouts, with or without light exposure. However, the differences in the nutrient contents between yellow and green alfalfa sprouts have not been reported in detail. The sequenced autotetraploid alfalfa germplasm “Zhongmu No. 1” was used for this study (Shen et al., 2020). Transcriptomic sequencing and broadly targeted metabolomic sequencing of its yellow and green alfalfa sprouts were performed to better understand the mechanisms underlying different nutrient profiles of alfalfa sprouts. This work will provide a reference for consumer selection and future research aimed at modulating the levels of specific nutrients in alfalfa sprouts.

## Materials and methods

### Plant materials and growing conditions

Alfalfa (*Medicago sativa* L., cultivar “Zhongmu No. 1”) seeds were soaked for 12 h in the dark and then spread in a box made of filter paper. Each paper box was placed in a black plastic tray with grooves. Water was poured into the bottom of the tray to maintain the water level at the top of the grooves. For germination in the dark, the tray was covered with aluminum foil; for light germination, the tray was placed under an LED panel with a white light intensity of 30.8 μmol⋅m^–2^⋅s^–1^. A constant temperature of 20 ± 1°C was maintained, and the tray was refilled with water once daily. The weight and morphology of sprouts grown for different periods in the dark and light were characterized. We assessed seven different combinations of dark and light exposure. In the germination process, “0 + 6” represented 6 days of light, “1 + 5” indicated 1 day of darkness followed by 5 days of light, “2 + 4” represented 2 days of darkness and 4 days of light, “3 + 3” indicated 3 days of darkness followed by 3 days of light, “4 + 2” denoted 4 days of darkness followed by 2 days of light, “5 + 1” represented 5 days of darkness followed by 1 day of light, and “6 + 0” meant 6 days of darkness. Finally, yellow alfalfa sprouts grown for 6 days in the dark, green alfalfa sprouts grown for 4 days in the dark followed by 2 days of light exposure (16 h light/8 h dark), and alfalfa seeds soaked for 12 h were sampled for subsequent transcriptomic and metabolomic analyses. The material used for each analysis had at least three biological replicates, with each replicate pooled from at least 50 sprouts or soaked seeds (≥1 g). All data were presented as the mean ± standard deviation (SD) and were analyzed to show significant differences by ANOVA using GraphPad Prism 8.

### Transcriptomic analysis

Total RNA was extracted from frozen ground samples. The mRNA was enriched, fragmented, and reverse-transcribed into cDNA. The cDNA library construction and subsequent sequencing were performed at Metware Co., Ltd.^1^ The cDNA library quality was assessed with Qubit 2.0, Agilent 2100, and qRT-PCR. The Illumina HiSeq platform was used for sequencing. Clean reads were obtained after raw data filtering, sequencing error rate checks, and GC content distribution checks. Then, clean reads were aligned to the reference genome using HISAT2 to obtain the position information on the reference genome as well as the specific sequence characteristic information of the sequenced samples. Mapped reads were used to count the reads on the gene of each sample, and then the gene counting results of all the samples were assembled. DESeq2 was used to analyze differentially expressed genes (DEGs). Kyoto Encyclopedia of Genes and Genomes (KEGG^2^), Gene Ontology (GO^3^), and Clusters of Orthologous Groups of proteins (COG^4^) were used for functional annotation and enrichment analysis of DEGs.

### Metabolomic analysis

The metabolites present in the samples were determined using a widely targeted metabolomics analysis at Wuhan MetWare Biotechnology Co., Ltd. (see text footnote 1). Briefly, after vacuum freeze-drying, each sample was ground to a powder using a powered grinder (MM 400, Retsch) at 30 Hz for 1.5 min. A 100 mg sample was dissolved in 70% methanol extract, and the supernatant was absorbed and analyzed using ultra-performance liquid chromatography (UPLC; SHIMADZU Nexera X2) and a tandem mass spectrometry (MS/MS) system (Applied Biosystems 4500 QTRAP). Subsequently, principal component analysis (PCA) of samples and Pearson correlation coefficients (PCC) between samples were determined using R.^5^ Significantly regulated metabolites between groups were determined by variable importance of projection (VIP) ≥ 1 and absolute log_2_FC (fold change) ≥ 1. VIP values were extracted from the OPLS-DA results generated using the R package MetaboAnalystR. The Metware database (MWDB) and public databases were used to annotate metabolites. GO and KEGG analyses were used to identify differentially accumulated metabolites (DAMs).

### Quantitative real-time polymerase chain reaction

Ten genes were selected to verify RNA-Seq results by qRT-PCR. All gene-specific primers were designed using Primer 5.0 software and are listed in Supplementary Table 1. The same RNA samples used in RNA-Seq were used for qRT-PCR analysis. The cDNA was synthesized using HiScript^®^ III RT SuperMix (Vazyme #R323). qRT-PCR was performed using the ChamQ™ SYBR Color qPCR Master Mix (Vazyme #Q411) and run on the CFX96 qPCR system (Bio-Rad Laboratories, Shanghai, China). Relative expression was calculated using the 2^–ΔCT^ method. The data for gene expression are presented as the mean ± SD and were analyzed to detect significant differences by ANOVA using GraphPad Prism 8.

## Results and discussion

### Phenotypic characterization of alfalfa sprouts

**FIGURE 1:**
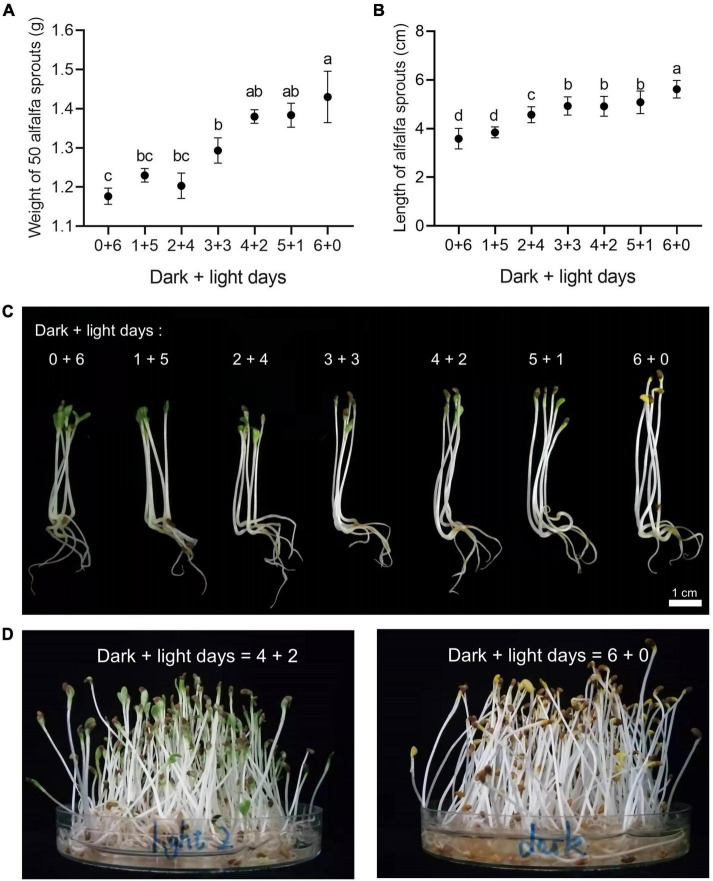
Effects of different dark and light days on alfalfa sprout morphology in a 6-day germination cycle. **(A)** Effects of different dark and light days on the weight of 50 alfalfa sprouts in a 6-day germination cycle. Different letters indicate statistically significant differences generated by ANOVA using GraphPad Prism 8; values are expressed as mean ± SD, *n* = 50, *P* ≤ 0.05. **(B)** Effects of different dark and light days on the length of alfalfa sprouts (including hypocotyl and cotyledon parts) in a 6-day germination cycle. Different letters indicate statistically significant differences generated by ANOVA using GraphPad Prism 8; values are presented as mean ± SD, *n* = 50, *P* ≤ 0.05. **(C)** Effects of different dark and light days on the morphology of alfalfa sprouts. **(D)** Green alfalfa sprouts germinated in 4 days of darkness and 2 days of light and yellow alfalfa sprouts germinated in 6 days of darkness were the samples selected for subsequent analysis.

A 6-day germination cycle with different combinations of days in the dark and under light exposure was applied to investigate the traits of the alfalfa sprouts (*Medicago sativa* L., cultivar “Zhongmu No. 1”). With the elongation of dark day incubation, the yields of alfalfa sprouts increased. In particular, the rate of alfalfa sprouts germination in the dark was significantly higher than that of sprouts germinated under light exposure for the same number of germination days (Figure 1A). Alfalfa sprouts germinating in the dark were longer than those germinating under light exposure (Figures 1B,C). There were no significant differences in the yield and length among 4 + 2 and 5 + 1 alfalfa sprouts (Figures 1A,B). Therefore, 6 + 0 alfalfa sprouts (yellow alfalfa sprouts) and 4 + 2 alfalfa sprouts (green alfalfa sprouts) were selected for subsequent transcriptomic and metabolomic analysis (Figure 1D).

In a soybean sprout study, the yield of green soybean sprouts was lower than that of yellow soybean sprouts (Chen and Chang, 2015), consistent with our observation of green and yellow alfalfa sprouts. Studies have found that cell wall thickness in tomato seedlings is significantly negatively correlated with hypocotyl growth. The cell wall is thicker under blue and white light exposure and thinner under far-red light and dark conditions (Falcioni et al., 2020). In our study, we found that the yellow alfalfa sprouts with longer hypocotyl were tenderer than the green alfalfa sprouts with shorter hypocotyl.

### Overall comparison of transcriptomic profiles between yellow and green alfalfa sprouts

**FIGURE 2:**
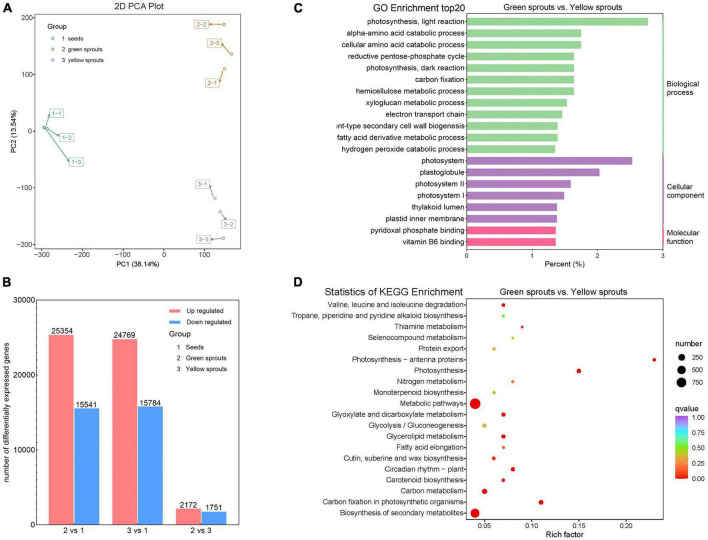
Transcriptomic analysis of alfalfa sprouts. **(A)** PCA score plot of transcriptome profiles from different treatment groups (1: 12-h-soaked alfalfa seeds; 2: green alfalfa sprouts; 3: yellow alfalfa sprouts). **(B)** Numbers of DEGs in 2 vs. 1, 3 vs. 1, and 2 vs. 3. **(C)** GO functional classification of DEGs between green and yellow sprouts. **(D)** KEGG pathway enrichment of DEGs between green and yellow sprouts.

To understand the molecular mechanisms of alfalfa sprouts, we performed RNA-Seq. A total of 80.66 Gb of sequencing data were generated from the nine strand–specific libraries. The number of clean reads for each sample ranged from 51.6 to 74.5 million, with a mean of 58.2 million. The Q30 values varied from 87.69 to 90.23%. High-quality reads were then aligned to the reference genome. All clean reads were compared to the reference genome of *Medicago sativa* L., and the ratio of successfully mapped reads ranged from 84.20 to 89.81%, with a mean of 87.02%. The count of mapped reads is summarized at the gene level in Supplementary Table 2. Correlation statistics between the transcriptome samples showed a high degree of consistency between different biological repetitions of the same germination regime, ensuring the reliability of the subsequent differential gene expression analysis (Supplementary Figure 1A). PCA of genes showed large and small differences between distinct germination regimes and biological repetitions of the same treatment, respectively (Figure 2A).

Differentially expressed genes for different comparisons were identified using DESeq2 based on FPKM (Fragments Per Kilobase of exon model per million mapped fragments) values. The expression levels of genes and the overall distribution of gene expression differences are shown in Supplementary Figures 1B–D and Supplementary Table 3. The numbers of DEGs identified in pairwise comparisons are presented in Figure 2B. The comparison between groups 2 (green alfalfa sprouts) and 1 (12-h-soaked alfalfa seeds) resulted in 40,895 DEGs, including 25,354 upregulated and 15,541 downregulated genes. The comparison between groups 3 (yellow alfalfa sprouts) and 1 (12-h-soaked alfalfa seeds) revealed a total of 40,553 DEGs, among which 24,769 were upregulated and 15,784 were downregulated. The comparison of groups 2 (green alfalfa sprouts) and 3 (yellow alfalfa sprouts) resulted in 3,923 DEGs, including 2,172 upregulated and 1,751 downregulated genes. These results indicated that, in terms of the number of DEGs, there were more significant differences between soaked seeds and different sprouts than green vs. yellow alfalfa sprouts.

In a pairwise comparison among the three groups, GO term enrichment analysis showed that “photosynthesis, light reaction” was the most abundant GO term among biological processes and that “photosystem” was the most abundant GO term among cellular components (Figure 2C; Supplementary Figures 2A,B). Comparing yellow and green alfalfa sprouts, DEGs were more enriched in “plastoglobule,” “photosystem II,” “photosystem I,” and “thylakoid lumen” in terms of cellular components (Figure 2C).

Kyoto Encyclopedia of Genes and Genomes enrichment analysis revealed that DEGs were significantly enriched in “metabolic pathways” and “biosynthesis of secondary metabolites.” DEGs also showed enrichment in photosynthesis-related pathways, such as “photosynthesis,” “photosynthesis-antenna proteins,” and “carbon fixation in photosynthetic organisms” (Figure 2D; Supplementary Figures 3A,B). When comparing yellow and green alfalfa sprouts, some DEGs were enriched in “carotenoid biosynthesis.” In plants, carotenoids help capture light and provide photoprotection and signal control over gene expression.

Photosynthesis requires the coordination of approximately 3,000 kinds of proteins. To carry out photosynthesis, plants require a large number of genes for the construction, replication, maintenance, and operation of chloroplasts (Kim et al., 2016; Wang et al., 2017). Wang et al. (2020) extracted, sequenced, and annotated the total RNA from etiolated (dark-grown) and green (light-grown) sprouts of Chinese fir and found that DEGs were mainly enriched in flavonoid biosynthesis, photosynthesis-antenna proteins, photosynthesis, cutin, suberine, wax biosynthesis, stilbenoid, diarylheptanoid, and gingerol biosynthesis. These findings are consistent with the results of our study. Grandellis et al. conducted research on potato buds under long-term darkness and long-term light exposure and found that under light conditions, the photosynthetic mechanism was fully activated, and the Rubisco activase (RCA), glyceraldehyde-3-phosphate dehydrogenase (GAPDH), and Photosystem II 22 kDa protein (CP22) genes were the most upregulated. Seedlings continuously grown in the dark showed longer elongation times. After prolonging the dark period, the synthesis of chloroplast components was inhibited (Grandellis et al., 2016). Light drives the expression of specific proteins. For instance, the alfalfa-plastocyanin protein found in light-grown alfalfa sprouts is only expressed in the leaves and seedlings of alfalfa plants grown under light exposure, and its promoter region contains light-responsive cis-acting elements (Su et al., 2013). In our results, for both GO term and KEGG pathway analyses, green alfalfa sprouts expressed more photosystem-related genes than yellow alfalfa sprouts (Figures 2C,D). Accordingly, the genes coding plastocyanin protein had higher expression levels in green than yellow alfalfa sprouts (Supplementary Table 3).

Seed germination involves several biochemical changes. During the germination process, hydrolytic enzymes, such as proteins, polysaccharides, and fat, are activated to hydrolyze macromolecules, resulting in an increase in the content of oligopeptides, free amino acids, monosaccharides, oligosaccharides, and fatty acids, ultimately promoting plant growth and increasing plant utilization of nutrients. For consumers, the germination process reduces or eliminates various anti-nutritional factors in the seeds, such as tannins, phytic acid, and protease inhibitors, thereby improving the digestibility of nutrients and utilization of vitamins and minerals. The germination process also produces a variety of biologically active substances such as vitamins, γ-aminobutyric acid, and polyphenols (Gan et al., 2017; Saithalavi et al., 2021). In our study, thousands of genes corresponded with seed germination are expressed in green or yellow alfalfa sprouts (Supplementary Table 3).

### Overall comparison of metabolites between yellow and green alfalfa sprouts

To better understand the nutrient composition and nutritional value of alfalfa sprouts, we performed a metabolite analysis of alfalfa sprouts. A total of 818 metabolites were detected in all three groups (Supplementary Table 4). The PCA results shown in Supplementary Figure 4A, especially the distinguishing metabolite grouping of different sample groups, enabled the next step in the metabolomic analysis. Among these, 21 metabolites were present only in soaked alfalfa seeds; 39 metabolites in both yellow and green alfalfa sprouts; one metabolite in both soaked seeds and yellow alfalfa sprouts; 757 metabolites were found in all groups. These 818 metabolites were classified into 13 known classes, including 183 flavonoids, 139 lipids, 105 phenolic acids, 83 organic acids, 83 amino acids and derivatives, 55 terpenoids, 52 nucleotides and derivatives, 51 saccharides and alcohols, 29 alkaloids, 16 vitamins, 10 lignans and coumarins, three tannins, and one stilbene; eight remaining metabolites with unknown classifications were assigned to the “others” group (Supplementary Figure 4B). The metabolite composition did not significantly differ between green and yellow alfalfa sprouts, except for isoquercitrin, which was present in yellow alfalfa sprouts and soaked alfalfa seeds but absent in green alfalfa sprouts (Supplementary Table 4).

**FIGURE 3:**
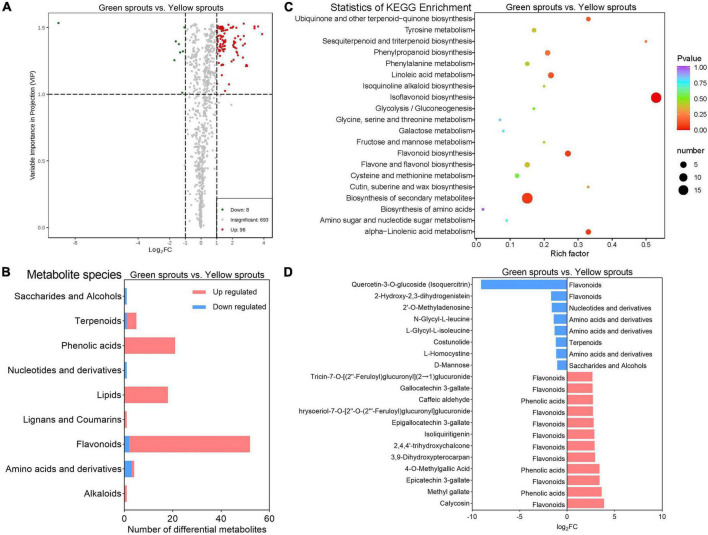
Metabolomic analysis of alfalfa sprouts. **(A)** DAM volcano map of green vs. yellow alfalfa sprouts. **(B)** The number of DAMs that were up- and downregulated in different classes between green and yellow alfalfa sprouts. **(C)** KEGG pathway enrichment of DAMs between green and yellow alfalfa sprouts. **(D)** The eight downregulated DAMs and the 12 most upregulated DAMs in green vs. yellow alfalfa sprouts.

Differences in the levels of metabolite expression between the three groups of samples and the statistical significance of the differences are shown in the volcanic plot (Figure 3A; Supplementary Figures 4C,D). A total of 504, 485, and 104 DAMs were identified when comparing green alfalfa sprouts vs. soaked alfalfa seeds (2 vs. 1), yellow alfalfa sprouts vs. soaked alfalfa seeds (3 vs. 1), and green alfalfa sprouts vs. yellow alfalfa sprouts (2 vs. 3), respectively. Moreover, the number of upregulated DAMs was higher than that of downregulated DAMs in the above three group comparisons.

Differentially accumulated metabolites were sorted into their corresponding classes to investigate the metabolic characteristics of each group (Figure 3B; Supplementary Figures 5A,B). Comparing groups 3 vs. 1 and 2 vs. 1 showed similar metabolic characteristics. Primary metabolites in the vitamin class and most of the primary metabolites in the amino acids and derivatives, nucleotides and derivatives, organic acids, saccharides, and alcohols were upregulated in groups 2 and 3 compared to those in group 1. Primary metabolites in lipids were upregulated in group 2 compared to group 1, whereas some lipids were downregulated in group 3 compared to group 1. Secondary metabolites in the lignan and coumarin classes and most of the secondary metabolites in the alkaloids and terpenoid classes were upregulated in groups 2 and 3 compared to group 1. Secondary metabolites in the tannins and most secondary metabolites in the flavonoids were downregulated in groups 2 and 3 compared to group 1. Most of the secondary metabolites in phenolic acids were upregulated in group 2 compared to group 1, while there were more downregulated than upregulated phenolic acids in group 3 than in group 1. When comparing groups 3 and 2, no significant differences were observed among organic acids, tannins, and vitamin classes. All lipids and most flavonoids, phenolic acids, and terpenoids were upregulated in group 2 relative to group 3.

Phenolic compounds, widely present in plants, affect color, aroma, and taste. They are also beneficial to human health and exert antioxidant and anti-inflammatory effects. Phenolic compounds, a heterogeneous group of phytochemicals comprising phenol rings bearing one or more hydroxyl groups, include flavonoids, phenolic acids, tannins, stilbenes, anthocyanins, xanthines, and lignans. Terpenoids are important secondary metabolites in plants. Their basic backbone structure is synthesized by terpene synthase (TPS) and further modified by hydroxylation, dehydrogenation, acylation, or glycosylation, resulting in an array of chemically diverse terpenoid compounds (Abbas et al., 2017). Terpenoids help pollination, participate in plant defense reactions, and help control pests, weeds, and pathogenic bacteria. For consumers, terpenoids have not only antibacterial and antioxidant effects but also have special pharmacological activities (Bergman et al., 2019).

Light is a critical environmental factor for plants, as it not only provides an energy source for photosynthesis but also initiates a variety of plant physiological reactions, promoting plant growth. Light can stimulate nutrient production and accumulation in plants, improving crop quality. Studies have found that light activates the biosynthesis and metabolism of sterol lipids, prenol lipids, and polyunsaturated lipids required for photosynthesis in broccoli sprouts, increasing flavonoid levels (Maldini et al., 2015). Under light conditions, the total phenolic and flavonoid contents in pea sprouts are upregulated (Liu et al., 2016). In our study, the accumulation of lipids, phenolic acids, and flavonoids was also observed in light-exposed green alfalfa sprouts when compared with yellow alfalfa sprouts (not exposed to light).

Kyoto Encyclopedia of Genes and Genomes enrichment analysis was conducted to analyze the DAMs in pathways. The results showed that the biosyntheses of secondary metabolites were significantly enriched in alfalfa sprouts compared to seeds (Supplementary Figures 5C,D). When comparing yellow and green alfalfa sprouts, “isoflavonoid biosynthesis,” “flavonoid biosynthesis,” “alpha-linolenic acid metabolism,” “linoleic acid metabolism,” and “phenylpropanoid biosynthesis” were significantly enriched (Figure 3C). Phenylpropanoid metabolism is one of the most important metabolic pathways in plants. Its first three steps constitute the widely reported general phenylpropanoid pathway, which is connected to primary and secondary metabolism and provides precursors for all downstream metabolites. Flavonoid metabolism is an important component of phenylpropane metabolism. Flavanones synthesized *via* this pathway are the starting point for isoflavonoid biosynthesis. In most plants, the major unsaturated fatty acids (UFAs) are three C18 species, namely oleic (18:1), linoleic (18:2), and α-linolenic (18:3) acids. C18 UFAs can be used as intrinsic antioxidants, jasmonic acid precursors, and extracellular barrier components. C18 UFAs participate in plant stress responses and play a regulatory role in plant defenses. At the same time, two polyunsaturated fatty acids, linoleic and α-linolenic acids, are essential dietary fatty acids for humans (He et al., 2020).

There were 96 upregulated DAMs and eight downregulated DAMs in groups 2 vs. 3 (Supplementary Table 5). Eight downregulated DAMs and the top 12 upregulated DAMs were identified (Figure 3D). Yellow alfalfa sprouts contained more isoquercitrin, and green alfalfa sprouts consisted of a wide variety of flavonoids and phenolic acids such as calycosin, methyl gallate, and epicatechin 3-gallate. Isoquercitrin could remove reactive oxygen/nitrogen species and exert antioxidant activity and chemical protection against oxidative stress, cancer, cardiovascular disease, diabetes, and allergic reactions. Isoquercitrin can egress plasma and tissues in an intact form after oral administration due to its good water solubility and high bioavailability (Valentova et al., 2014). Calycosin is a phytoestrogen with anti-cancer, anti-inflammatory, anti-osteoporosis, neuroprotective, and liver-protecting pharmacological properties (Deng et al., 2021).

### Metabolic pathways regulated at the transcriptional level

**FIGURE 4:**
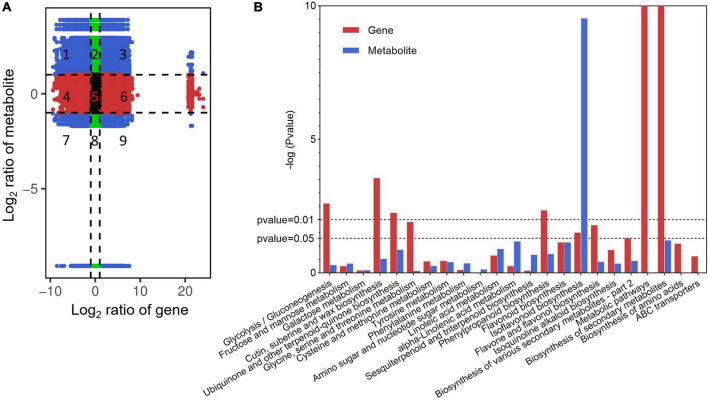
Profiles of the association analysis of genes and metabolites in green vs. yellow alfalfa sprouts. **(A)** Nine-quadrant map of genes and metabolites. **(B)** KEGG enrichment analysis of differential metabolites and genes between green and yellow alfalfa sprouts. The *x*-axis represents the metabolic pathway. The *y*-value represents the -log (*P*-value) of differentially expressed genes (DEGs) and differentially accumulated metabolites (DAMs).

Correlation analysis was conducted for genes and metabolites detected in each group. The genes and metabolites with PCC greater than 0.8 in each group were displayed through a nine-quadrant graph (Figure 4A). The third and seventh quadrants represent genes and metabolites with a consistent regulatory trend, respectively, and the change in metabolites may be positively regulated by genes. The first and ninth quadrants represent genes and metabolites with inconsistent regulatory trends, and the change in metabolites may be negatively regulated by genes.

Based on the KEGG enrichment analysis of differential metabolites and genes, a histogram was drawn to simultaneously depict the enrichment degree of the pathway with DAMs and DEGs between groups 2 and 3 (Figure 4B). Results showed that DEGs and DAMs enriched in the isoflavonoid biosynthesis pathway exhibited consistent expression patterns with statistically significant differences (*P* < 0.05).

**FIGURE 5:**
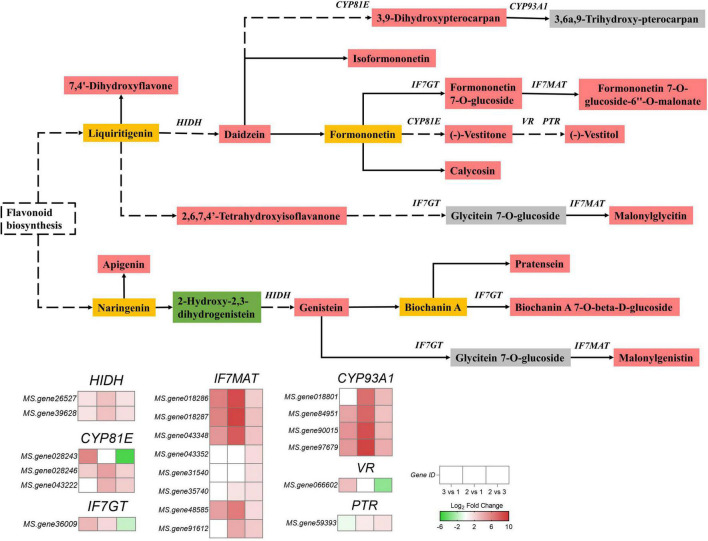
Expression profiles of genes and metabolites involved in isoflavonoid biosynthesis of green vs. yellow alfalfa sprouts (2 vs. 3). The rectangle patterns of different colors represent metabolite accumulations in comparison 2 vs. 3: red indicates upregulated metabolites; green shows downregulated metabolites; yellow indicates that metabolites were detected, but there was no difference in their expression, and gray stands for metabolites that were undetected. The different colored squares indicate gene expression levels in comparisons 3 vs. 1, 2 vs. 1, and 2 vs. 3. Red and green represent up- and down-regulated genes, respectively.

A total of 20 genes and 17 metabolites were enriched in the isoflavonoid biosynthesis pathway with a consistent or inconsistent regulatory trend (Supplementary Table 6). We constructed a pathway diagram that included the expression heat map of each DEG in the isoflavonoid biosynthesis pathway of the alfalfa sprouts (Figure 5). Compared with group 3, most DEGs were upregulated in group 2.

Two 2-hydroxyisoflavanone dehydratase genes (*HIDH*) (*MS.gene26527* and *MS.gene39628*) were upregulated. The isoflavone 7-*O*-glucosyltransferase gene (*IF7GT*) (*MS.gene36009*) was downregulated. Two isoflavone 2’-hydroxylase genes (*CYP81E*) (*MS.gene028246*, *MS.gene043222*) were upregulated, but one, *CYP81E* (*MS.gene028243*), was downregulated. Eight isoflavone 7-*O*-glucoside-6″-*O*-malonyltransferase genes (*IF7MAT*) (*MS.gene018286*, *MS.gene018287*, *MS.gene043348*, *MS.gene043352*, *MS.gene31540*, *MS.gene35740*, *MS.gene48585*, and *MS.gene91612*) were upregulated. A vestitone reductase gene (*VR*) (*MS.gene066602*) was downregulated, and the pterocarpan reductase gene (*PTR*) (*MS.gene59393*) was upregulated. Four 3,9-dihydroxypterocarpan 6a-monooxygenase genes (*CYP93A1*) (*MS.gene018801*, *MS.gene84951*, *MS.gene90015*, and *MS.gene97679*) were upregulated.

Isoflavones are a class of phenolic compounds mainly found in Leguminosae, including isoflavones, pterocarpans, coumestan, and other subclasses (Veitch, 2007). Isoflavonoids can be used as signal molecules to promote symbiosis between plants and nitrogen-fixing bacteria and are also plant defensins that participate in the inhibition of pathogenic bacteria. Isoflavonoids scavenge free radicals and are positively associated with human health by reducing the risk of hormone-dependent cancers, menopausal symptoms, osteoporosis, and cardiovascular diseases (Dastmalchi et al., 2017; Han et al., 2017).

In this study, green alfalfa sprouts accumulated more isoflavonoids than yellow alfalfa sprouts, indicating that light affects isoflavonoid accumulation in alfalfa sprouts. Aisyah et al. (2013) showed that light exposure during soybean germination also increased the total isoflavonoid content.

### Quantitative real-time polymerase chain reaction analysis

**FIGURE 6:**
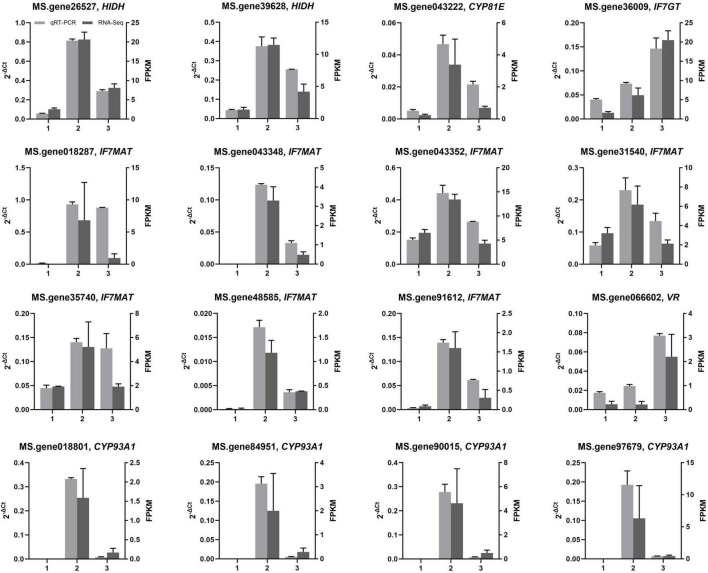
Analysis of gene expression related to the isoflavonoid biosynthesis pathway. Detection of the relative expression of selected genes in alfalfa seeds (1), green (2), and yellow (3) alfalfa sprouts by RNA-seq and qRT-PCR; values are expressed as means ± SD.

To validate the accuracy and repeatability of transcriptome analysis in the present study, qRT-PCR was conducted on a set of DEGs associated with isoflavonoid biosynthesis. Among all candidate DEGs related to isoflavonoid biosynthesis, 16 were selected for qRT-PCR analysis. Figure 6 illustrates the expression of all 16 selected DEGs as determined by qRT-PCR and RNA-Seq. Overall, the expression trends of the 16 DEGs, as determined by qRT-PCR, were consistent with the corresponding FPKM values derived from RNA-Seq analysis. The core genes for isoflavonoid biosynthesis, such as *HIDH*, *IF7MAT*, and *CYP93A1*, were more significantly expressed in green than yellow alfalfa sprouts, matching our observation of isoflavonoid accumulation in green alfalfa sprouts compared with yellow alfalfa sprouts (Figures 5, 6).

## Conclusion

Our study revealed the physiological and biochemical changes during the alfalfa germination process, as well as the metabolism and gene expression changes in alfalfa sprouts when exposed to light. The germination process involves changes in a variety of plant genes and metabolites, and differential genes are significantly enriched in the establishment of photosynthesis-related structures and the photosynthetic process. The germination process also involves the metabolism of various amino acids, carbohydrates, lipids, cofactors, vitamins, terpenoids, and polyketides, as well as the biosynthesis of other secondary metabolites. The yellow alfalfa sprouts germinated in the dark, and the light-exposed green alfalfa sprouts were relatively different in terms of flavonoid, phenolic acid, and lipid composition. Green alfalfa sprouts have a higher content of isoflavonoids and UFAs, whereas yellow alfalfa sprouts have a higher content of isoquercitrin. In the conjoint analysis of transcription and metabolism, we found that DAMs and DEGs were significantly enriched in the flavonoid biosynthesis pathway and the content of isoflavones such as daidzein, genistein, and calycosin increased. The differential expression of six key genes in this pathway, namely *HIDH*, *HI4OMT*, *IF7GT*, *IF7MAT*, *CYP81E*, and *CYP93A1*, was identified. Our analysis revealed differences in quality between green and yellow alfalfa sprouts, providing a reference for future research and alfalfa sprout production.

## Data availability statement

The original contributions presented in the study are publicly available. This data can be found here: https://www.ncbi.nlm.nih.gov/, PRJNA838779.

## Author contributions

KZ, HX, Z-YW, and MC designed the research. KZ, HL, TZ, SW, LL, XD, LC, HS, AW, GY, and HX performed the experiments and analyzed the data. KZ, HX, and MC wrote the manuscript. All authors contributed to the article and approved the submitted version.

## Footnotes

1
www.metware.cn

2
https://www.genome.jp/kegg

3
http://www.geneontology.org/

4
http://clovr.org/docs/clusters-of-orthologous-groups-cogs/

5
www.r-project.org

## Supplementary material

Supplementary Figure 1 Examination of differentially expressed genes (DEGs) in alfalfa seeds and sprouts. **(A)** Correlation heat map of different treatment groups determined by Pearson’s correlation coefficient. **(B)** MA (ratio intensity) map of DEGs of green alfalfa sprouts vs. soaked alfalfa seeds. **(C)** MA (ratio intensity) map of DEGs of yellow alfalfa sprouts vs. soaked alfalfa seeds. **(D)** MA (ratio intensity) map of DEGs of green alfalfa sprouts vs. yellow alfalfa sprouts.

Supplementary Figure 2 GO enrichment analysis of DEGs in alfalfa seeds and sprouts. **(A)** GO functional classification of DEGs in green alfalfa sprouts vs. soaked alfalfa seeds. **(B)** GO functional classification of DEGs in yellow alfalfa sprouts vs. soaked alfalfa seeds.

Supplementary Figure 3 KEGG pathway analysis of DEGs in alfalfa seeds and sprouts. **(A)** KEGG pathway enrichment of DEGs in green alfalfa sprouts vs. soaked alfalfa seeds. **(B)** KEGG pathway enrichment of DEGs in yellow alfalfa sprouts vs. soaked alfalfa seeds.

Supplementary Figure 4 DAM analysis in alfalfa seeds and sprouts. **(A)** PCA score plot of metabolite profiles from different treatment groups. **(B)** Classification and the number of all detected metabolites. **(C)** DAM volcano map of green alfalfa sprouts vs. soaked alfalfa seeds. **(D)** DAM volcano map of yellow alfalfa sprouts vs. soaked alfalfa seeds.

Supplementary Figure 5 KEGG pathway analysis of DAMs in alfalfa seeds and sprouts. **(A)** The number of up- and downregulated DAMs in different classes in green alfalfa sprouts vs. soaked alfalfa seeds. **(B)** The number of up- and downregulated DAMs in different classes in yellow alfalfa sprouts vs. soaked alfalfa seeds. **(C)** KEGG pathway enrichment of DAMs in green alfalfa sprouts vs. soaked alfalfa seeds. **(D)** KEGG pathway enrichment of DAMs in yellow alfalfa sprouts vs. soaked alfalfa seeds.
