# Supplementary figures and images for: Comprehensive transcriptomic and metabolomic profiling reveals the differences between alfalfa sprouts germinated with or without light exposure
Source: Front Plant Sci. 2022 Aug 5;13:943740. doi: 10.3389/fpls.2022.943740 (PMC9389271; doi:10.3389/fpls.2022.943740)

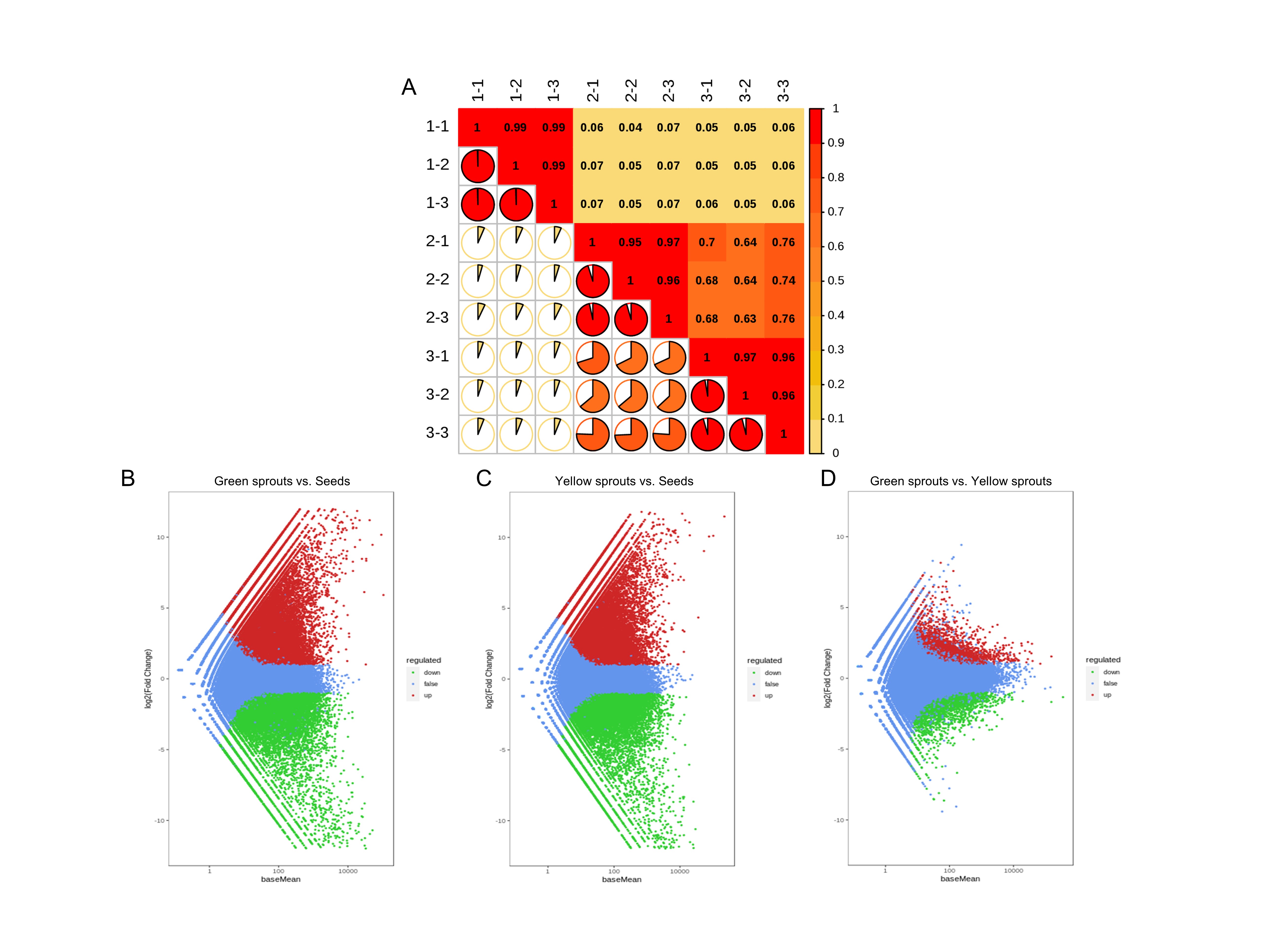

Supplement: Supplementary Figure 1 — Examination of differentially expressed genes (DEGs) in alfalfa seeds and sprouts. (A) Correlation heat map of different treatment groups determined by Pearson’s correlation coefficient. (B) MA (ratio intensity) map of DEGs of green alfalfa sprouts vs. soaked alfalfa seeds. (C) MA (ratio intensity) map of DEGs of yellow alfalfa sprouts vs. soaked alfalfa seeds. (D) MA (ratio intensity) map of DEGs of green alfalfa sprouts vs. yellow alfalfa sprouts. [file Image_1.JPEG]

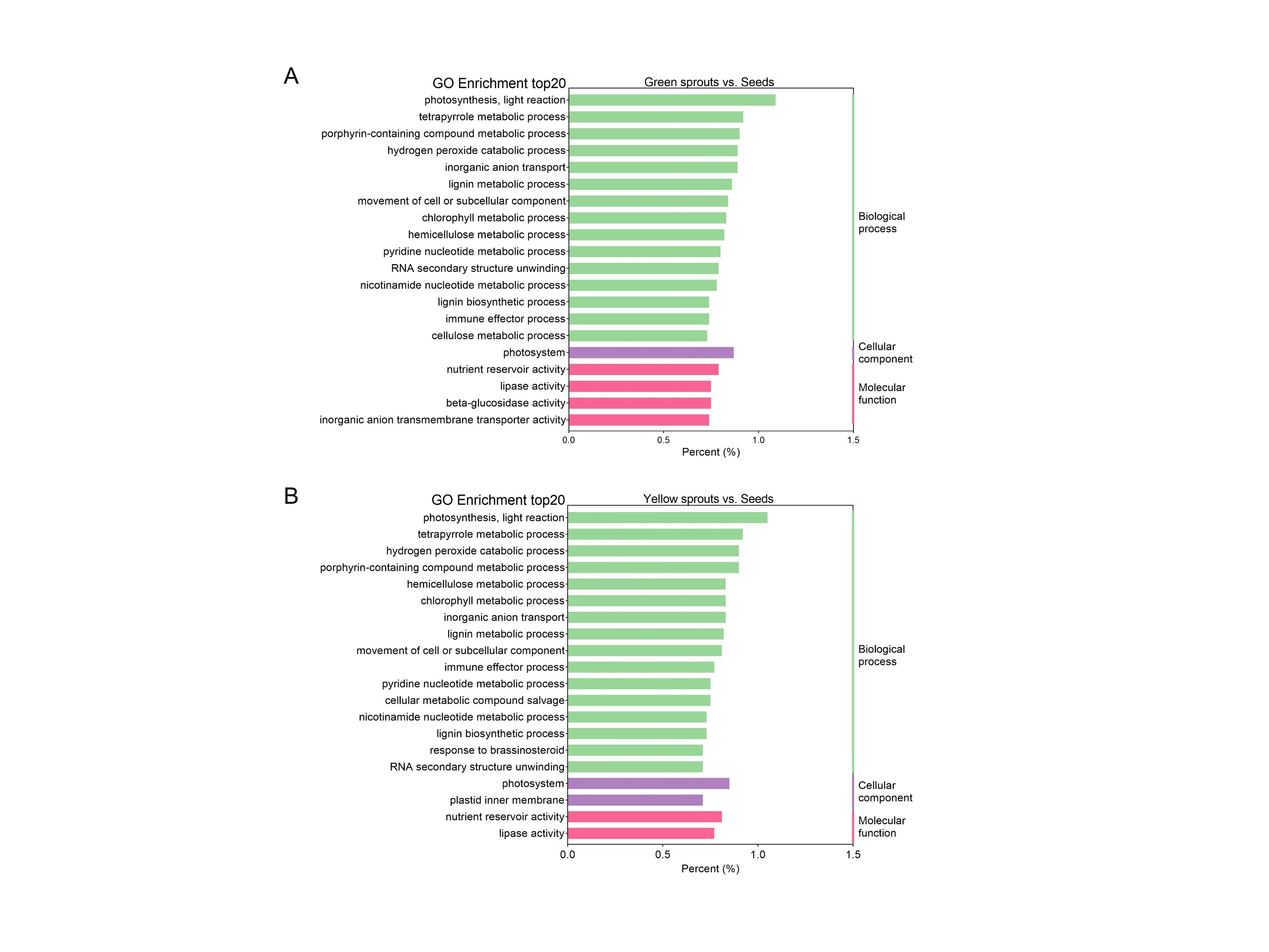

Supplement: Supplementary Figure 2 — GO enrichment analysis of DEGs in alfalfa seeds and sprouts. (A) GO functional classification of DEGs in green alfalfa sprouts vs. soaked alfalfa seeds. (B) GO functional classification of DEGs in yellow alfalfa sprouts vs. soaked alfalfa seeds. [file Image_2.JPEG]

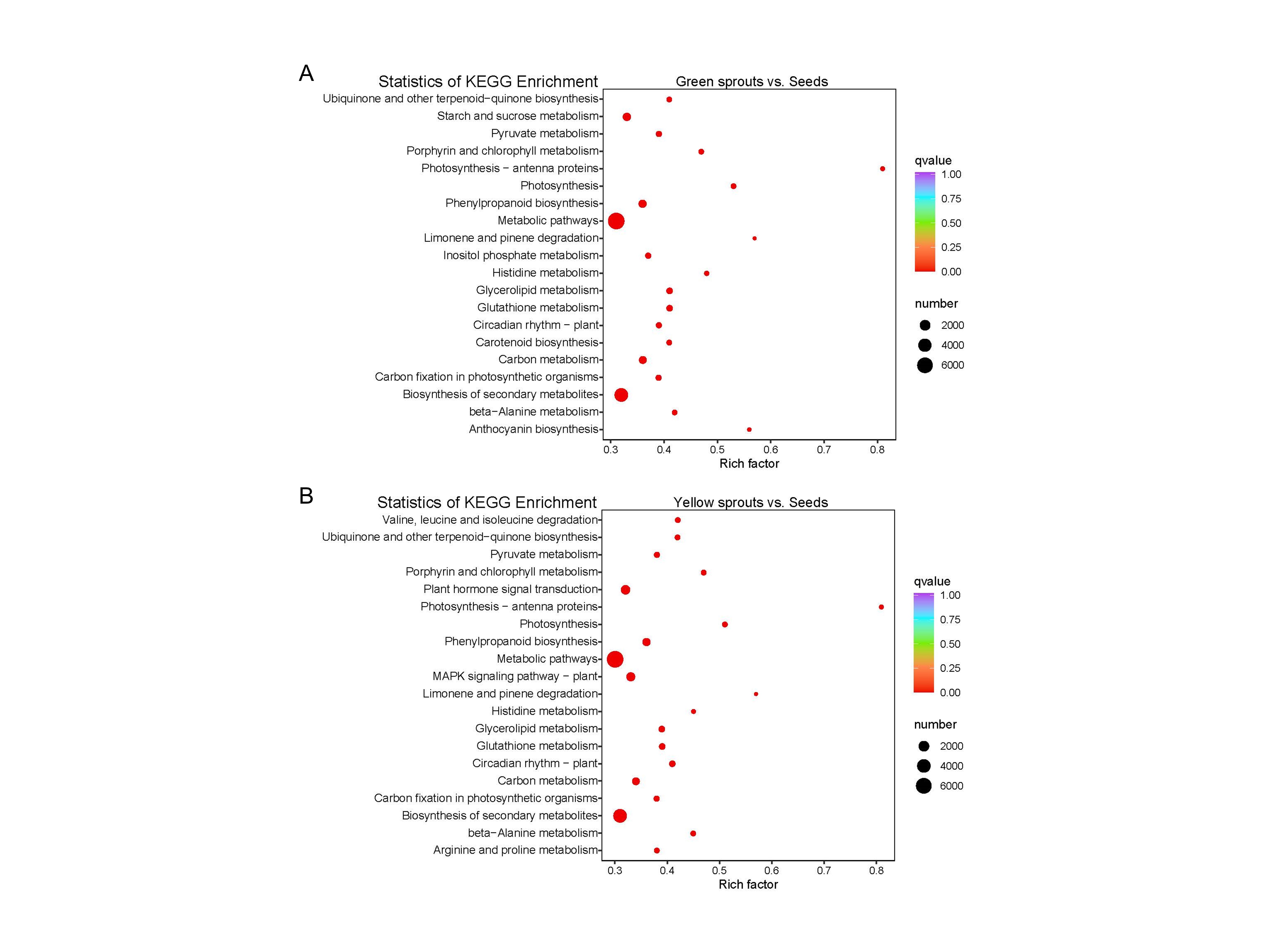

Supplement: Supplementary Figure 3 — KEGG pathway analysis of DEGs in alfalfa seeds and sprouts. (A) KEGG pathway enrichment of DEGs in green alfalfa sprouts vs. soaked alfalfa seeds. (B) KEGG pathway enrichment of DEGs in yellow alfalfa sprouts vs. soaked alfalfa seeds. [file Image_3.JPEG]

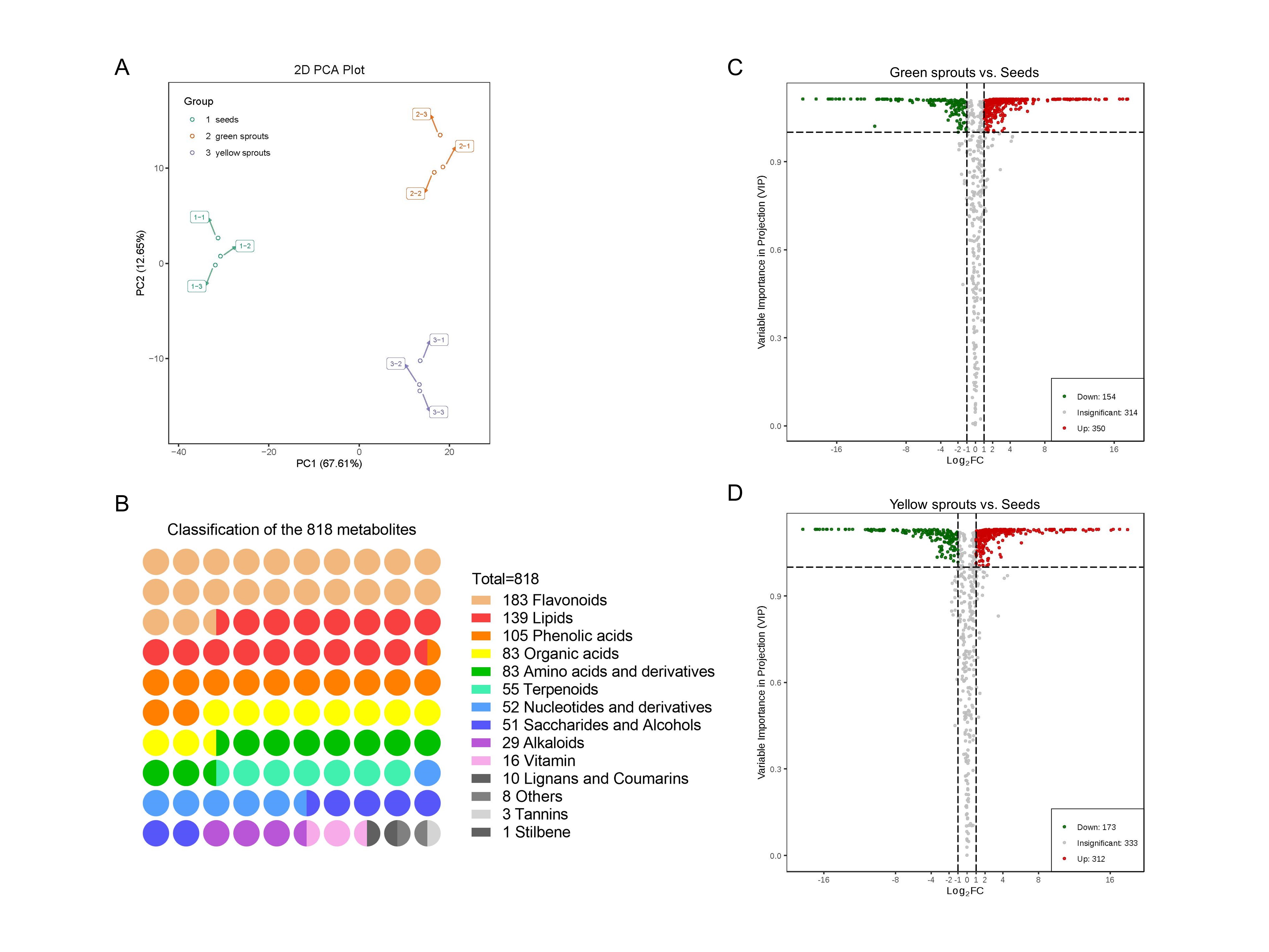

Supplement: Supplementary Figure 4 — DAM analysis in alfalfa seeds and sprouts. (A) PCA score plot of metabolite profiles from different treatment groups. (B) Classification and the number of all detected metabolites. (C) DAM volcano map of green alfalfa sprouts vs. soaked alfalfa seeds. (D) DAM volcano map of yellow alfalfa sprouts vs. soaked alfalfa seeds. [file Image_4.JPEG]

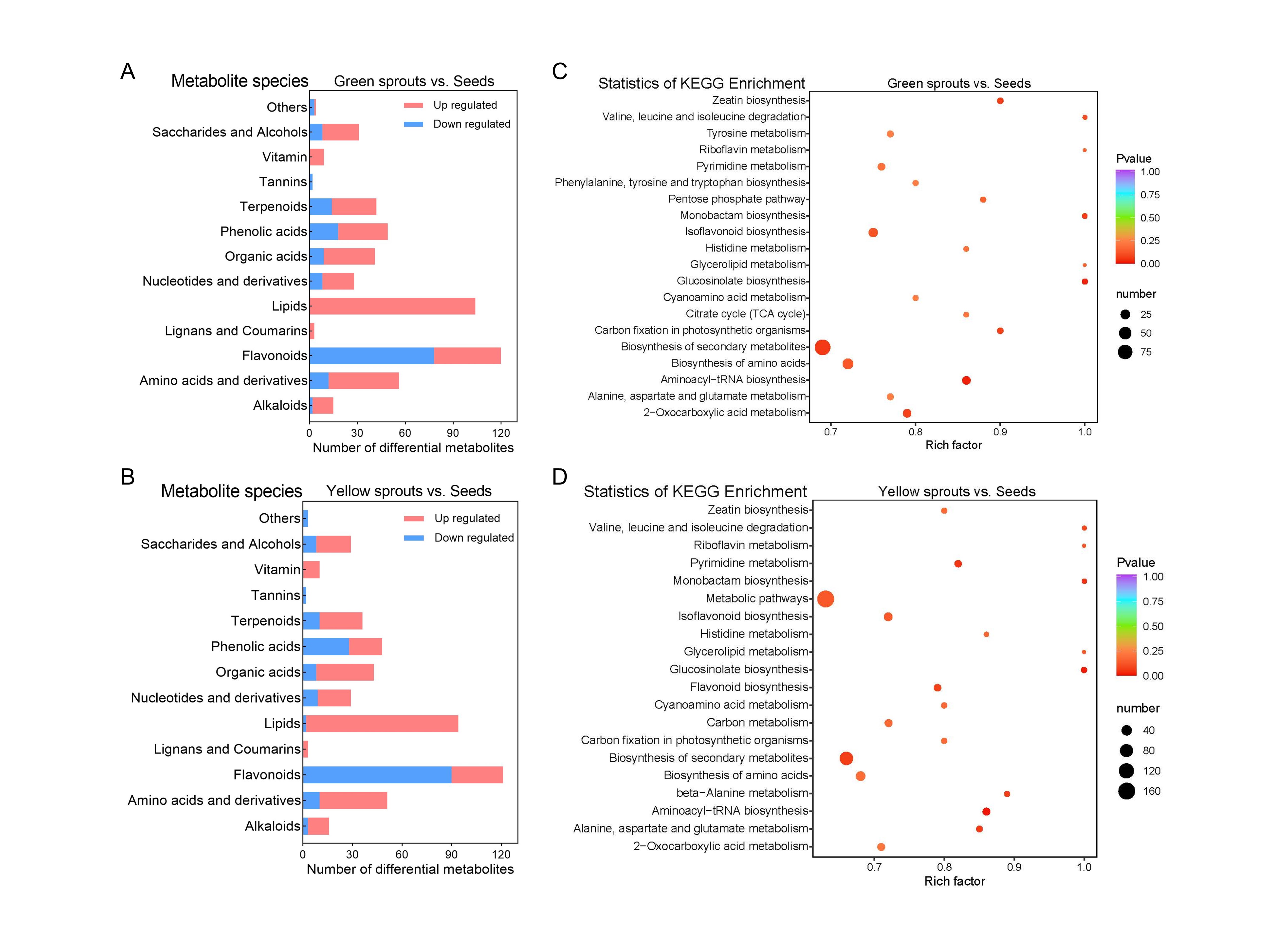

Supplement: Supplementary Figure 5 — KEGG pathway analysis of DAMs in alfalfa seeds and sprouts. (A) The number of up- and downregulated DAMs in different classes in green alfalfa sprouts vs. soaked alfalfa seeds. (B) The number of up- and downregulated DAMs in different classes in yellow alfalfa sprouts vs. soaked alfalfa seeds. (C) KEGG pathway enrichment of DAMs in green alfalfa sprouts vs. soaked alfalfa seeds. (D) KEGG pathway enrichment of DAMs in yellow alfalfa sprouts vs. soaked alfalfa seeds. [file Image_5.JPEG]
